# Egg Removal via Cloacoscopy in Three Dystocic Leopard Geckos (*Eublepharis macularius*)

**DOI:** 10.3390/ani13050924

**Published:** 2023-03-03

**Authors:** Alessandro Vetere, Enrico Bigliardi, Marco Masi, Matteo Rizzi, Elisa Leandrin, Francesco Di Ianni

**Affiliations:** 1 Department of Veterinary Science, University of Parma, Strada del Taglio 10, 43126 Parma, Italy; 2 Centro Veterinario Specialistico, Via Sandro Giovannini, 51/53, 00137 Roma, Italy; 3 Clinica Veterinaria Madonna Di Rosa, Via Rosa 1, 33078 San Vito Al Tagliamento, Italy

**Keywords:** *Eublepharis macularius*, dystocia, endoscopy, reproductive disorders

## Abstract

**Simple Summary:**

Three adult female leopard geckos (*Eublepharis macularius*) belonging to three different owners were referred to for coelomic distention, anorexia, and weight loss. X-rays showed the presence of a macrosomic egg set in the third caudal of the coelomic cavity, and the diagnosis of dystocia was made in all three geckos. A cloacal endoscopic examination was performed on all three animals. A voluminous egg protruding through the urogenital papilla to the cloaca was visible. All the eggs were removed easily using endoscopic forceps. In two geckos, the eggshell was torn, and the content was aspirated to reduce the egg volume. After the procedure, a subcutaneous deslorelin implant was implanted. All geckos recovered rapidly after surgery. Two of the three geckos were healthy at the follow-up visit (respectively one and two years after the surgery) and did not show any signs of dystocia. Only in the third gecko, the dystocia recurred again 6 months later, and bilateral ovariosalpingectomy was performed. After surgery, the gecko recovered rapidly, resumed eating, and was discharged after one week of hospitalization in good condition.

**Abstract:**

Dystocia is a multifactorial, life-threatening condition commonly affecting pet reptiles. Treatment for dystocia can be either medical or surgical. Medical treatment usually involves the administration of oxytocin, but in some species or, in some cases, this treatment does not work as expected. Surgical treatments such as ovariectomy or ovariosalpingectomy are resolutive, but invasive in small-sized reptiles. In this paper, we describe three cases of post ovulatory egg retention in three leopard geckos (*Eublepharis macularius*) successfully treated through a cloacoscopic removal of the retained eggs, after a non resolutive medical treatment. The intervention was fast, non-invasive, and no procedure-related adverse effects were noted. The problem relapsed six months later in one animal, and a successful bilateral ovariosalpingectomy was performed. Cloacoscopy should be considered a valuable, non-invasive tool for egg removal in dystocic leopard geckos when the egg is accessible to manipulation. Recrudescence or complications such as adhesions, oviductal rupture, or the presence of ectopic eggs should recommend surgical intervention.

## 1. Introduction

Dystocia (egg binding) in reptiles is a common, multifactorial disease [1]. Different factors of captivity are often related to the occurrence of dystocia, such as inadequate husbandry (improper temperature gradients or humidity levels, inadequate nesting sites, overcrowding), poor physical conditions (illness, dehydration, malnutrition), reproductive apparatus disorders (infectious diseases, traumatic injuries, misshapen or large eggs), and metabolic diseases (hypocalcemia secondary to nutritional or renal hyperparathyroidism) [1,2]. Two common forms of reproductive disorders in reptiles are recognizable: preovulatory follicular stasis and postovulatory stasis or egg binding. The first form occurs when egg development stops prior to ovulation after vitellogenesis, resulting in persistent follicles and leading to inflammation and eventually rupture, coelomitis, and death [2,3,4]. The second form, the postovulatory stasis, occurs when the eggs are not laid and retained inside the oviduct for an indefinite period of time, or ectopic [1,3,4,5]. In both conditions, the animal can refuse to eat and its conditions can deteriorate rapidly, leading it to death [4,5]. The diagnosis of postovulatory stasis is usually made by X-ray [4,6,7] due to the radiopacity of the eggshell. Retained eggs can become overcalcified and appear more radiopaque on radiographic images [6]. Egg presence can also be assessed through careful palpation of the mid- or caudal coelom. Vitellogenic follicles during preovulatory stasis are better visualized and even measured through ultrasound or CT scan [8,9,10,11]. Treatment for dystocia can be either medical or surgical. Medical treatment involves the administration of oxytocin (1 to 10 UI/kg) intramuscularly (IM) or the administration of arginine vasotocine [12,13]. Oxytocin seems to work better in chelonians than in snakes and lizards [12]. It is generally used one hour after IM calcium gluconate administration, especially if the blood calcium level is low [12,14]. Arginine vasotocine seems to be more effective in reptiles than oxytocin, but the short storage life, high cost, and unlicensed use make this medical treatment option problematic in clinical practice [12]. Percutaneous ovocentesis is often reported by some authors as a treatment of dystocia in geckos [7]. However, it carries a high risk of organ rupture, with consequent coelomitis. If the eggs can be visualized through the cloacal opening, ovocentesis can be performed to make the eggs collapse and therefore be removed more easily [3]. Hormonal therapy, such as deslorelin implants, seems to be ineffective in suppressing ovarian activity in leopard geckos [15,16] and its use is still under debate in other reptiles [17,18,19]. In cases where medical treatment fails or is not applicable, surgery remains the elective choice. Ovariectomy or ovariosalpingectomy is resolutive [4,5,20].

## 2. Clinical Cases

### 2.1. Histories and Clinical Examination

#### 2.1.1. Case 1

A 2-year-old, 45 g captive bred female leopard gecko (*Eublepharis macularius*) was presented for clinical examination due to one week of anorexia and weight loss. The animal was kept in a 60 (length) × 50 (height) × 30 (depth) cm glass terrarium without substrate, with only paper towels at the bottom that were changed weekly. The temperature inside the cage was 30 °C (86 °F) during the day and 25 °C (77 °F) at night. The diet was consistent and consisted of insects with calcium powder given three times a week. A UVB light bulb with 5.0 spectrum was provided and changed every 6 months. A plastic box with peat moss was present in the terrarium and misted daily. The owner indicated that the gecko was acquired two weeks earlier from another breeder. Once introduced in the new environment, the gecko spent the most of time burrowed in the substrate. At clinical examination, the gecko was lethargic and mildly to moderately dehydrated. The coelomic cavity was distended, and the presence of a large egg was palpable and visible in transparency through the skin in the caudal half of the coelom on the right half of the sagittal plane.

#### 2.1.2. Case 2

**Figure 1:**
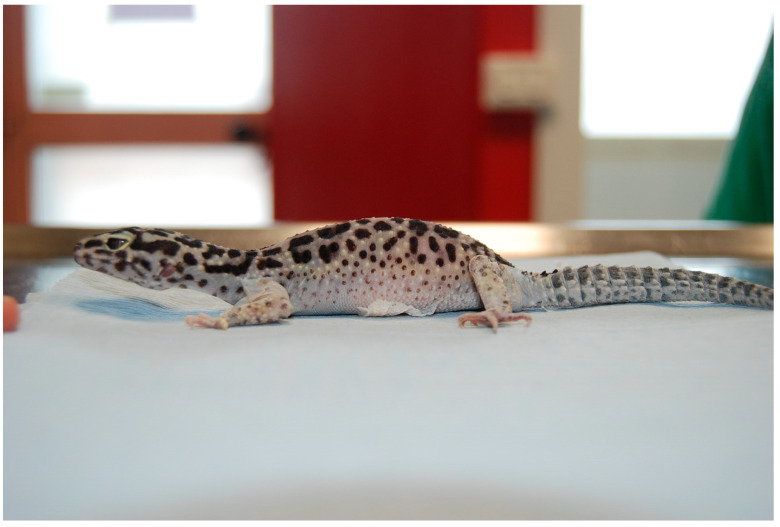
Case 2: The gecko presented with distenction of the coelomic cavity, dysecdysis, and poor nutritional status.

A 4-year-old, 50 g captive bred female leopard gecko (*Eublepharis macularius*) was presented for clinical examination due to dysecdysis, lethargy, anorexia, and distention of the coelomic cavity (Figure 1). The animal was housed with a two-year-old male in a 50 (length) × 50 (height) × 50 (depth) cm glass terrarium with paper towels as a substrate and maintained at 30 °C (86 °F) during the day and 24 °C (75.2 °F) at night. The diet was consistent and consisted of insects dusted with calcium powder given twice a week. A UVB light bulb with 5.0 spectrum was provided and changed every 6 months. The owner also reported that a box with a wet mixture of peat moss and coconut fiber was added to the terrarium to facilitate deposition, and the female spent most of the time burrowing inside the box. No eggs were found during the daily check by the owner. At clinical examination, the gecko was lethargic and moderately dehydrated. The abdomen was swollen, and the presence of two voluminous masses was palpable in the caudal half of the coelom on the left and right halves of the sagittal plane.

#### 2.1.3. Case 3

A 4-year-old, 52 g captive bred female leopard gecko (*Eublepharis macularius*) was presented for a veterinary second opinion. The owner indicated that the gecko was kept with an adult male of unknown age; both were kept in an 80 (length) × 40 (height) × 40 (depth) glass terrarium with paper towels as a substrate at 32 °C (89.6 °F) at day and 24 °C (75.2 °F) at night. In the cage, a box with a wet mixture of peat moss and coconut fiber was present as a wet nest to facilitate molting, and after the suggestion of the original breeder, deposition. The female spent most of the time burrowing inside without eating for two weeks. The diet was consistent and consisted of insects dusted with calcium powder given twice a week. No UVB light was provided. The animal was first presented due to lethargy and anorexia, and a diagnosis of egg binding was made by the first veterinarian based on the anamnesis and by the dorsoventral X-ray taken the same day. Oxytocin at 10 UI/kg was administered by the previous veterinarian. IM was performed twice with a one-hour interval without any results. At clinical examination, the gecko was lethargic and mildly dehydrated. The coelomic cavity was distended, and the presence of a large egg was palpable in the caudal half of the coelom on the right half of the sagittal plane.

### 2.2. Diagnostic Procedures

#### 2.2.1. Case 1

**Figure 2:**
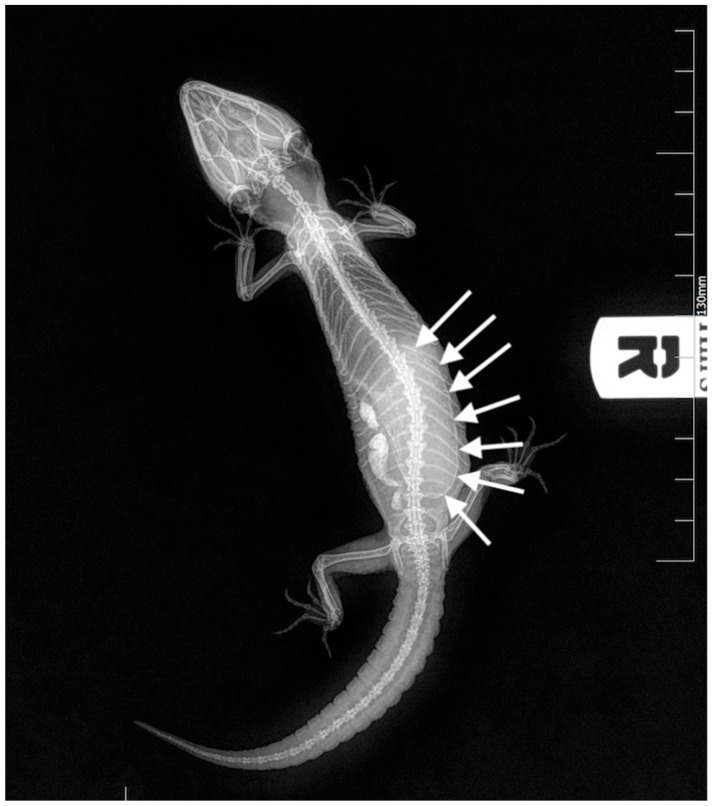
Case 1: Dorso-ventral X-ray. A large egg was visible in the caudal coelom (white arrows).

**Figure 3:**
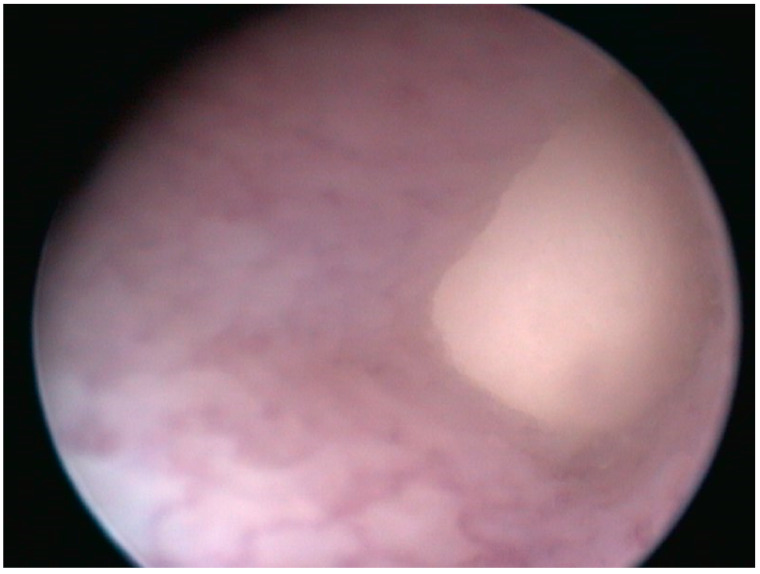
Case 1: Cloacoscopy. Endoscopic view of the right egg protruding from the oviduct to the cloaca.

**Figure 4:**
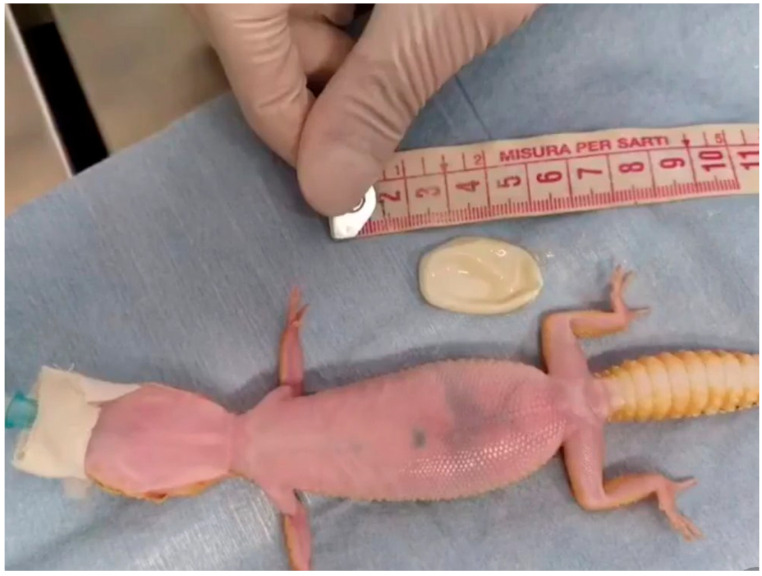
Case 1: End of the procedure. The egg was aspirated and easily extracted through the cloacal opening.

Complete blood work, X-rays, and ultrasounds were performed. The CBC count and PCV were unremarkable. The biochemistry tests showed a moderate increase in AST and the CK X-rays showed the presence of large eggs in the caudal half of the coelomic cavity on the right half of the sagittal plane, and the presence of radiopaque foreign material was interpreted as ingested sand in the intestinal tract (Figure 2). Ultrasound examination confirmed the egg presence and the presence of hyperechoic material inside the intestinal lumen; no oviduct rupture was noted. Oxytocin at 5 UI/kg (10 UI/mL neurofisin, FATRO S.p.a, Ozzano dell’Emilia, Italy) [14] was administered in the right tricep after IM administration of calcium gluconate at 100 mg/kg (200 mg/mL Calcio PH, FATRO S.p.a, Ozzano dell’Emilia, Italy) 1 h before, without success. Given the inefficacy of the treatment, cloacoscopy was planned to examine any potential oviduct abnormalities and determine whether the egg could be removed through endoscopic grasping forceps, avoiding a more invasive celiotomy. The animal was anaesthetized using alfaxalone (10 mg/mL Alfaxan, Dechra Veterinary Products Srl, Torino, Italia) at a dosage of 5 mg/kg delivered intravenously in the right jugular vein [21]. The heart rate and respiratory rate were monitored during all procedures. The animal lost the rightening reflex after approximately 40 s but experienced apnea, so it was intubated and mechanically ventilated (one breath every 10 s). A 2.7 mm × 18 cm, 30° oblique telescope (within a 4.8 mm operative sheath) (Storz Telepack TP100 EN, Karl Storz Endoscopia Italia Srl, Verona, Italy) was used for cloacal inspection, and showed one egg protruding from the right salpinx to the cloaca (Figure 3). During the procedure, the operator held the animal in ventral recumbency with the left hand, while the right hand maneuvered the telescope. The eggshell was broken using grasping forceps, and the content was removed without any difficulties (Figure 4, Video S1).

#### 2.2.2. Case 2

**Figure 5:**
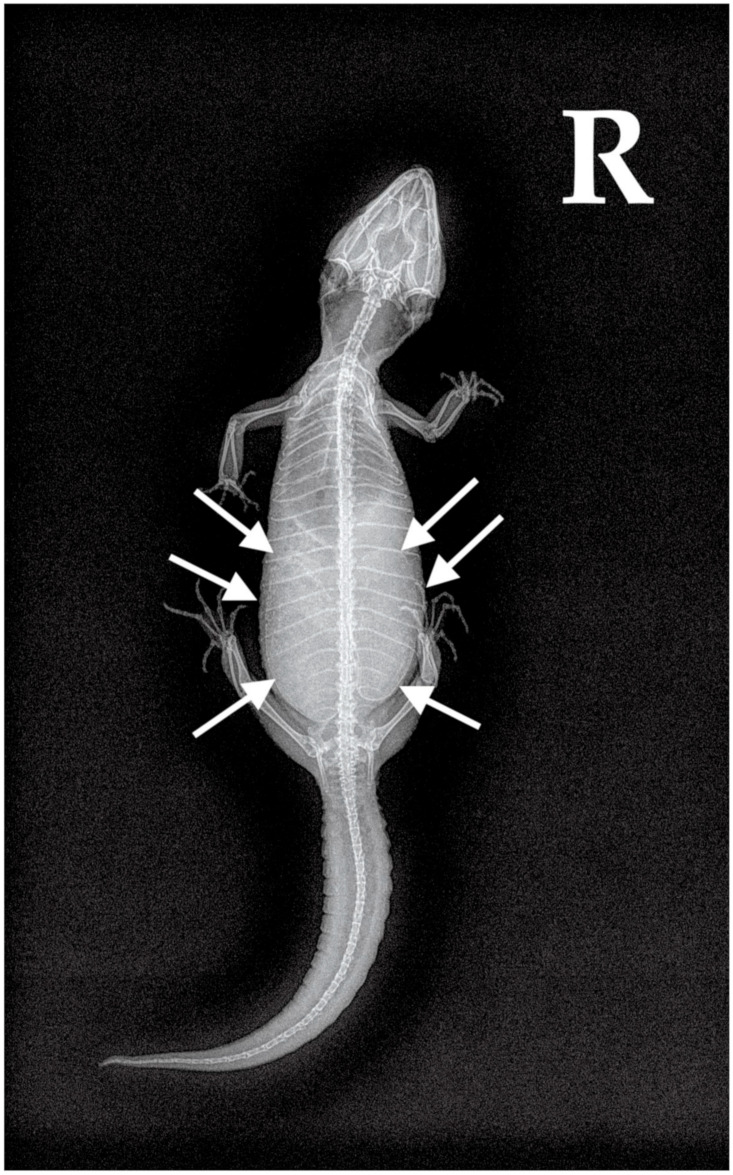
Case 2: Dorsoventral X-ray. Two large eggs were visible in the caudal coelom (white arrows).

**Figure 6:**
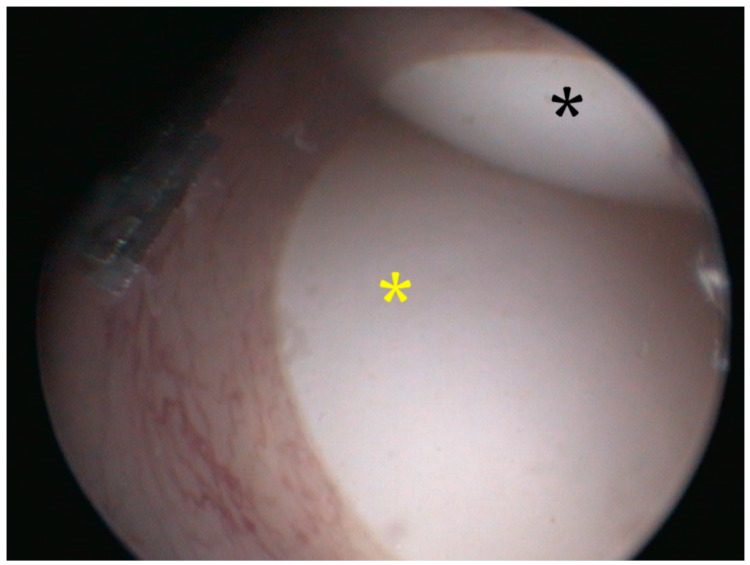
Cloacoscopy. Endoscopic view of the right (black asterisk) and the left (yellow asterisk) egg protruding from the vagina to the cloaca.

Complete blood work, X-rays, and ultrasounds were performed. The CBC count showed an increased WBC count with moderate heterofilia and monocytosis, mild basophilia, mild eosinopenia, and lymphocytosis (Table 1). The PCV was mildly increased. Biochemistry tests showed a moderate increase in creatinine kinase (Table 2). X-rays were performed in dorsoventral and latero-lateral projections, showing the presence of two large eggs in the caudal coelom (Figure 5). Ultrasound examination confirmed the presence of eggs; no oviduct rupture was noted. The use of oxytocin as medical treatment was not performed due to the severe weakness of the gecko. Due to the mild dehydration, warm fluids (ringer solution) were administered subcutaneously at 10 mL/kg/die and stabilized at 28 °C for 12 h. Cloacoscopy was planned for the day after to verify whether the eggs were visible and removable using endoscopic grasping forceps, avoiding a more invasive celiotomy. The animal was anesthetized using alfaxalone (10 mg/mL Alfaxan, Dechra Veterinary Products Srl, Via Agostino da Montefeltro, 2, Torino, Italia) at a dosage of 5 mg/kg delivered intravenously in the right jugular vein [21]. The animal lost the rightening reflex after approximately 60 s and maintained spontaneous breathing during the entire procedure. A 2.7 mm × 18 cm, 30° oblique telescope (within a 4.8mm operative sheath (Storz Telepack TP100 EN, Karl Storz Endoscopia Italia Srl, Verona, Italy) was used for cloacal inspection and showed the surface of the two eggs protruding from the left and right salpinx to the cloaca (Figure 6). During the procedure, the operator held the animal in ventral recumbency with the left hand, while the right hand maneuvered the telescope, as described in the Case 1. The eggshell was broken using grasping forceps, and the content was removed by performing cloacal lavages with warm sterile NaCl solution. The empty eggshells were removed as described for case 1.

#### 2.2.3. Case 3

**Figure 7:**
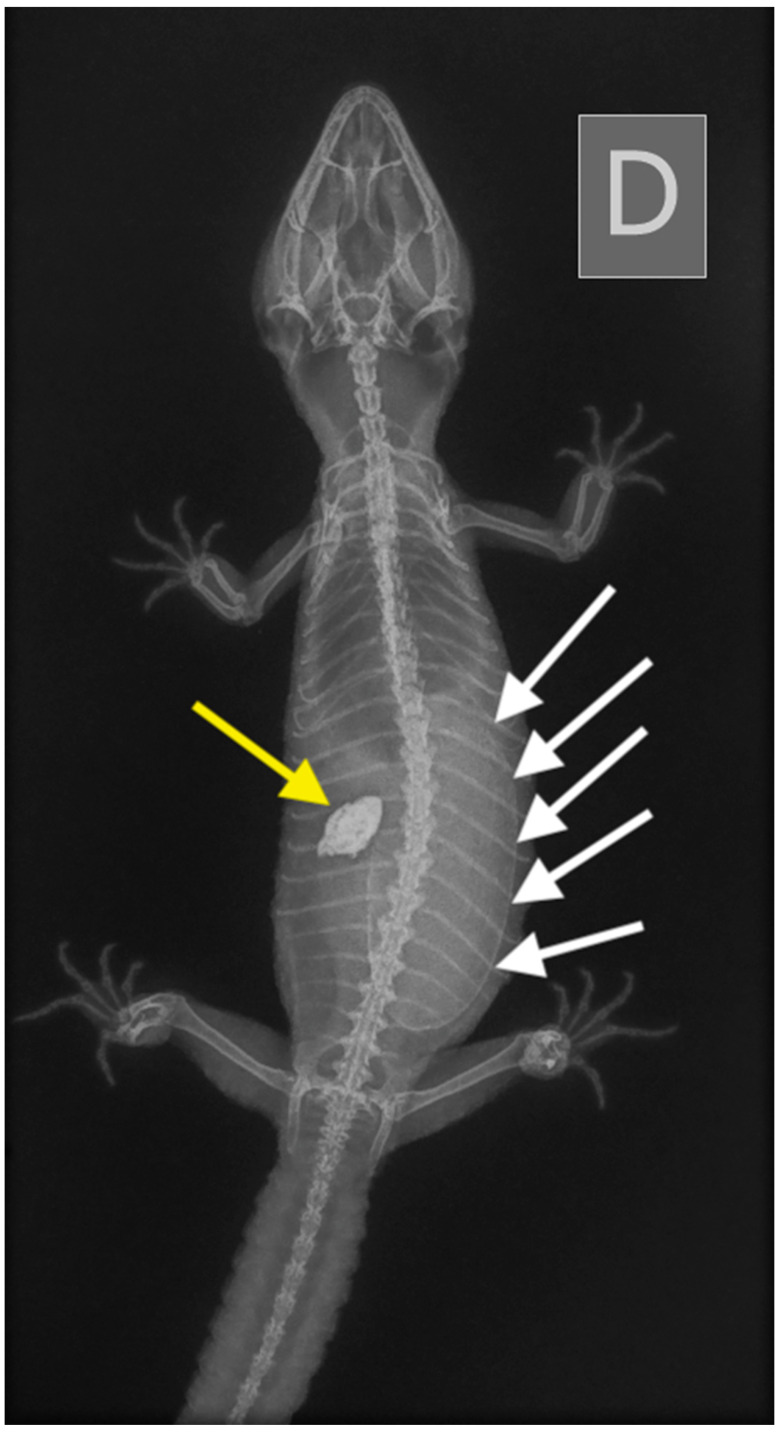
Case 3: Dorso-ventral X-ray. A large egg was visible in the caudal half of the coelomic cavity (white arrows).

Complete blood work, X-rays, and ultrasounds were performed. The CBC count showed a mildly increased WBC count with mild basophilia. The PCV was mildly increased. Biochemistry tests were unremarkable. X-rays were repeated and showed the presence of one large hypocalcified egg on the left half of the coelomic cavity (Figure 7) and a high radiopaque area on the left side, measuring approximately 0.5 cm × 1 cm in diameter. Ultrasound examination confirmed the egg presence in the right oviduct and the presence of a hyperechoic foreign body in the left oviduct. No oviduct ruptures were noted. Cloacoscopy was planned for the day after. As described for the case 2, warm fluids (ringer solution) were administered subcutaneously at 10 mL/kg/die and the gecko stabilized at 28 °C for 24 h. The animal was anesthetized using the same protocol described for cases 1 and 2. The animal lost the rightening reflex after approximately 40 s and maintained spontaneous breathing during the entire procedure. A 2.7 mm × 18 cm, 30° oblique telescope (within a 4.8 mm operative sheath) (Storz Telepack TP100 EN, Karl Storz Endoscopia Italia Srl, Verona, Italy) was used for cloacal inspection and showed the surface of the egg protruding from the right salpinx to the cloaca. During the procedure, the operator held the animal as described in case 1 and 2. The eggshell was broken, and the content was removed by performing cloacal lavages with warm sterile NaCl solution. The empty eggshells were removed as described for cases 1 and 2. A subcutaneous deslorelin implant (4.7 mg, Suprelorin^®^, Milano, Italy) was applied in the neck region upon the owner’s request despite the owner having been informed that there is no scientific evidence of its efficacy in leopard geckos.

### 2.3. Postoperative Care and Follow-Up

#### 2.3.1. Case 1

The gecko recovered 15 min after the end of the procedure, and no adverse effects were noted. Warm fluids (ringer solution) were administered subcutaneously at 15 mL/kg/die at the end of the endoscopic procedure to support the renal function after the anesthesia. Assisted feeding (Emeraid IC critical care formula, Emeraid LLC, A Division of Lafeber, Cornell, IL, USA) was administered once a day as nutritional support. Five days after the procedure, the gecko started to eat, so it was discharged. One month later, the gecko’s weight had increased by 5 g, and she was active and in good nutritional status. One year later, the gecko laid six infertile eggs, but dystocia had not occurred.

#### 2.3.2. Case 2

The gecko recovered 20 min after the end of the procedure, and no adverse effects were noted. Fluid therapy and nutritional support were administered as described in case 1. Two days after the procedure, the gecko started to eat, so it was discharged. The owner was instructed to separate the male gecko from the female gecko and the problem had never occurred at the two-year follow-up.

#### 2.3.3. Case 3

**Figure 8:**
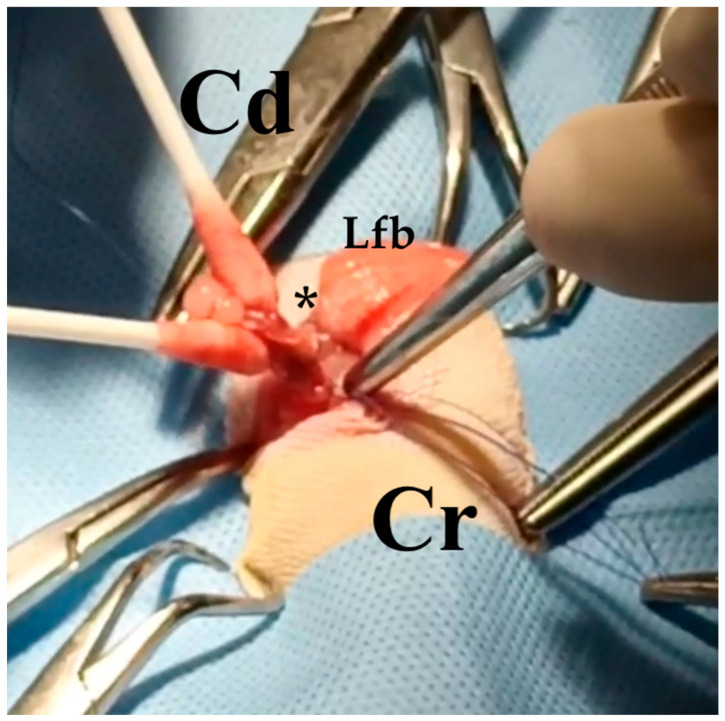
Case 3: Surgery. Left ovarian stalk (black asterisk) ligature during the ovariosalpingectomy intervention. Cr: cranial. Cd: caudal. Lfb: left fat body.

The gecko recovered 18 min after the end of the procedure, and no adverse effects were noted. Warm fluids (ringer solution) were administered subcutaneously at 15 mL/kg/die at the end of the endoscopic procedure. Fluid therapy and nutritional support were administered as described in case 1. Five days after the procedure, the gecko started to eat, so it was discharged. The owner was instructed to separate the male gecko from the female gecko. The leopard gecko did not lay any eggs in the next 6 months, and then a new episode of dystocia occurred; a large egg was evident in the right oviduct again, and the calcification in the left oviduct was still present. A bilateral ovariosalpingectomy was proposed and accepted by the owner. The gecko was premedicated with a mixture of dexmedetomidine at a dosage of 0.1 mg/kg (0.5 mg/mL Dexdomitor^®^, Vetòquinol Italia S.r.l, Bertinoro, Italy) and ketamine at a dosage of 10 mg/kg (100 mg/mL Ketavet^®^, Intervet production Srl, Aprilia, Italy) delivered in the right triceps [24]. The animal was intubated, maintained under gaseous anesthesia with 2% isoflurane (IsoFlo^®^, Zoetis Italia s.r.l, Roma, Italy) and mechanically ventilated (one breath every 15 s). A paramedian incision was performed to avoid the abdominal venous sinus. The cutis, abdominal muscles, and the coelomic membrane were cut, and the right oviduct with a voluminous egg inside was rapidly identified and isolated. A right salpingotomy was performed to remove the egg, making the access to the ovaries easier and improving the visibility of the near organs and blood vessels. Both the right and left ovaries and the respective oviducts were removed after ligature of the main vessels (Figure 8) with a 7/0 absorbable monofilament (Monosyn^®^ Braun Avitum Italy S.p.A. Mirandola, Italy). The hyperechoic structure visible on ultrasound examination revealed a residual hypercalcified eggshell inside the left salpinx. The coelomic membrane was closed with a simple continuous suture through the abdominal muscles with a 6/0 absorbable monofilament (Monosyn^®^ Braun Avitum Italy S.p.A. Mirandola, Italy). The cutis was closed with an everted simple interrupted suture with a 6/0 absorbable monofilament (Monosyn^®^ Braun Avitum Italy S.p.A. Mirandola, Italy). Atipamezole at a dosage of 0.5 mg/kg (Atidorm^®^ Fatro Industria Farmaceutica Veterinaria S.p. A, Ozzano dell’Emilia, Italy) was administered to the left triceps muscles at the end of the surgical procedure. The animal awakened 4 min after administration of the anesthesia reversal agent. A single 5 mg/kg dose of tramadol was administered IM, and 0.2 mg/kg meloxicam was administered SC once a day (SID) [25] postoperatively. The antimicrobial ceftazidime was administered SC every 3 days at a dose of 20 mg/kg [26]. The gecko started to eat after 2 days of therapy and was discharged after one week of hospitalization in good condition. At the 3-month follow-up, the clinical examination was unremarkable.

## 3. Discussion

Rigid endoscopy is a useful tool for the direct and also indirect evaluation of the reproductive tract in reptiles [3,27,28,29]. Coelioscopy is used as a direct method for the evaluation of the reproductive tract in chelonians, snakes, and lizards [3,28,29,30]. In tortoises, cystoscopy permits an indirect visualization of the coelomic organs in transparency through the urinary bladder wall [30,31]. This technique also permits the detection of retained eggs in the urinary bladder as well as their removal [32,33,34]. To the authors’ knowledge, this is the first report of endoscopic-assisted egg removal through cloacoscopy in leopard geckos. All three cases presented here were diagnosed as obstructive postovulatory stasis in three female leopard geckos. As stress can be a predisposing factor for dystocia [1,3], it is possible that the already pregnant female, recently acquired and recently housed in a new environment, developed postovulatory stasis due to a high level of stress. As sexually active males can attempt mating persistently, their constant presence in the same cage, together with the female, could have been a predisposing factor to dystocia in case 2 and case 3 [35,36].

In case 1, PCV% and CBC count were unremarkable. In case 2, PCV%, RBC, WBC, heterophils, basophils, monocytes, and lymphocytes were slightly increased (Table 1), and in case 3, PCV%, WBC, RBC and basophils were slightly increased. These alterations were considered to be related to the dehydration status [37] and not related to any reproductive disorder. In cases 1 and 2, CK was mildly augmented (Table 2). In reptiles, CK levels can vary with variations in environmental temperature [30]. Given the above, in case 1 and 2, the increase in CK was considered to not be related to any disease. AST, total protein, and uric acid were slightly increased in case 2 (Table 2). Increases in AST and total protein in reptiles with follicular development have been reported [30]; however, in this case, the mild increases in AST and total protein were thought to be related more to dehydration, as uric acid was also augmented. Oxytocin, as a promoter of muscle contractibility [14], was administered in cases 1 and 3 without success. It is possible that in geckos, oxytocin is less effective than in chelonians, as has been described for snakes and some lizards [12,14].

Deslorelin acetate is a synthetic nonapeptide analogue of the natural gonadotrophin-releasing hormone (GnRH). It acts as a contraceptive by temporarily suppressing the hypothalamic–pituitary–gonadal axis (HPG axis), inhibiting the production of pituitary hormones such as follicle-stimulating hormone (FSH) and luteinizing hormone (LH) [16,38,39]. In reptiles, the use of GnRH agonists has been poorly investigated; the use of deslorelin implants in leopard geckos seems to be less effective or ineffective at suppressing gonadal activity [15,16]. In lizards, suppression of gonadic activity was reported only in iguanas [38]. In chelonians, a desloreline implant was successfully used to treat chronic ovodeposition in a Greek tortoises (*Testudo graeca*) up to 24 months after application [40]. In a male *Chelonia mydas,* an annual application of deslorelin implants decreased serum testosterone levels after the fourth treatment [40].

In case 3, the gecko did not lay any eggs in the 6 months after treatment. A mature female leopard gecko can lay four to five clutches of two eggs per season with a one-month interval between clutches [36]. In this case, we do not know whether the gecko was at the end of the reproductive season or whether the ovarian activity was suppressed by the implant. Available scientific data [15] suggest that the latter hypothesis is unlikely. In all three cases, cloacal endoscopy permitted not only egg visualization but also egg removal through the cloacal opening under general anesthesia. Only one gecko (case 1) underwent apnea, and it was intubated and mechanically ventilated. Apnea has been reported in leopard geckos with the protocol used in case 1 [21]. In case 3, the recrudescence of egg binding required ovariosalpingectomy intervention. No procedure-related complications were noted.

The underlying causes of dystocia in these three cases remain unclear: it is likely that the voluminous eggs were too large to pass through the cloaca openings, or the oviduct was hypocontractile. Even the total calcium was considered normal according to the reference values [23], the ionized calcium was not measured. It is possible that an underlying hypocalcemia caused a decreased oviductal smooth muscle contractility [3,14]. The authors find this hypothesis unlikely since calcium supplementation was regularly provided by the owners. Moreover, no clinical signs of hypocalcemia such as seizures or skeletal deformities were noted at the physical examination. No evident fractures or skeletal demineralizations were radiographically detected. Diminishing egg pressure, leaking the shell with grasping forceps, and removal were performed easily. However, this procedure should be performed carefully to avoid the oviduct damage or rupture. A rude egg manipulation with the grasping forceps or a powerful cloacal flush could tear the oviductal or cloacal walls, predisposing to coelomitis.

Even if ultrasonography is a useful tool in diagnosing dystocia in reptiles [3,9,10], we were unable to visualize the distal part of the oviduct in all three geckos. In fact, the distal part of the reproductive tract is often difficult to image in lizards due to its location within the pelvic canal [41]. In these three described cases, the eggs protruding from the salpinx to the cloaca were visible and directly approachable during the cloacoscopy. This procedure should be critically evaluated whenever the eggs are not directly accessible or when adherences between the eggshell and the oviduct is suspected. Cloacoscopic egg removal should be considered before performing more invasive techniques, such as explorative celiotomy, when medical treatment fails. In fact, wound dehiscence and herniation are common post-surgical complication in leopard geckos, in relation to the thinness and the softness of the coelomic membrane and body wall musculature, which causes the muscles to tear more easily when tension is applied during the coelomic closure [33]. Percutaneous ovocentesis is contraindicated in reptiles and is not recommended because it increases the risk of yolk coelomitis and oviduct adherence. [9,28]. Many reptiles that are routinely presented for medical examinations are used for breeding and commercial purposes. In particular, leopard geckos have been bred for many years and today there are selections or “morphs” with a high commercial value on the market [42]. It is therefore of paramount importance to consider the owner’s desire to maintain functionality and the integrity of the reproductive system.

## 4. Conclusions

Considering the predisposition that leopard geckos have to dystocia, the use of the rigid endoscope as a minimally invasive instrument to reduce soft tissue trauma, duration, and post pain surgery, is a valuable option in all subjects affected by dystocia secondary to malformations of the egg, as in the cases described herein. Endoscopic egg removal could be a valuable tool in dystocic leopard geckos when the egg is accessible to manipulation, or in subjects in which it is essential to preserve the possibility of future reproduction. Adhesions, oviductal rupture, or the presence of ectopic eggs should recommend surgical intervention. However, the veterinarian must understand and correct the underlying causes of reproductive disorders to prevent a recurrence.

## Figures and Tables

**Table 1:** Comparison between CBC (complete blood count) parameters of the cases and the reference values. Abnormal values are in bold.

	Case 1	Case 2	Case 3	Normal Values [22,23] (Female)
PCV%	40	**45**	**45**	21–40%
WBC × 10^3^/mm^3^	8	**13**	**10**	6–9.4
RBC × 10^6^/mm^3^	0.7	**1.3**	**1.1**	0.43–0.89
Heterophils (10^3^/µL)	2.9	**3.5**	2	1.08–2.73
Eosinophils (10^3^/µL)	0.4	**0**	0.8	0.15–1.95
Basophils (10^3^/µL)	1	**3**	**3**	0.00–2.26
Monocytes (10^3^/µL)	1	**4**	2	0.60–2.16
Lymphocytes (10^3^/µL)	3	**4**	3	1.67–5.39

**Table 2:** Comparison between biochemical values of the cases and the reference values. Abnormal values are in bold.

	Case 1	Case 2	Case 3	Reference Values [22,23]
AST (U/L)	12	**78**		11–65
Total protein (g/dL)	7	**9**	4.2	2.4–8.0
Albumin	18	22	15	13–23
Creatinkinasis (U/L)	**4.897**	**4.000**	1861	0–3.701
Phosphorous (mg/dL)	14.5	8.4	14	1.5–16.4
Calcium (mg/dL)	18	22	31	14–>37
Potassium (mmol/lt)	6.8	5.5	6.1	4.50–7.0
Uric acid (mg/dL)	3.3	**6.7**	4	0.5–6.6 mg/dL
Biliary acids	0.6	3.2	1.1	0.8–21 μmol/L

## Data Availability

Data sharing is not applicable. No new data were created or analyzed in this study. Data sharing is not applicable to this article.

## Supplementary Materials

The following supporting information can be downloaded at: https://www.mdpi.com/article/10.3390/ani13050924/s1, Video S1.
